# Spontaneous Non-Traumatic Mediastinal Hematoma in a Patient on Imatinib Therapy for a Gastrointestinal Stromal Tumor (GIST)

**DOI:** 10.7759/cureus.37701

**Published:** 2023-04-17

**Authors:** Rida Aziz, Azeem Khan, Maryam Yousefi, Shivani Khetani, Hammad Choudhry

**Affiliations:** 1 Internal Medicine, William Carey University College of Osteopathic Medicine, Hattiesburg, USA; 2 Internal Medicine, Bayonne Medical Center, Bayonne, USA

**Keywords:** thoracic ct, spontaneous haemothorax, mediastinal hematoma, imatinib therapy, gastrointestinal stromal tumor (gist)

## Abstract

Mediastinal hematomas are thoracic complications often resulting from direct trauma or aortic dissections. Spontaneous non-traumatic mediastinal hematomas are rare. We present a case of spontaneous non-traumatic mediastinal hematoma in a patient on Imatinib therapy for a gastrointestinal stromal tumor (GIST). A 67-year-old female presented to the ER with the chief complaint of constant sharp right shoulder pain that progressed to her chest. The patient was not on any anticoagulants and had not complained of shortness of breath. Under suspicion of a pulmonary embolism, a CT chest scan was performed, and a diagnosis of non-traumatic anterior mediastinal hematoma was confirmed. This case may warrant further investigation into the links between Imatinib use and the formation of mediastinal hematomas.

## Introduction

Mediastinal hematomas are relatively common complications, often resulting from trauma or aortic dissection. However, spontaneous non-traumatic mediastinal hematomas are rare [1,2]. There have been reports of Imatinib therapy for gastrointestinal stromal tumors (GIST) linked with hepatic and subdural hematomas [3,4]. Imatinib is a tyrosine kinase inhibitor that has been shown to arrest or even reverse the progression of GIST [2,5]. Imatinib at 400 mg/day has now become part of the standard of care for treating GIST [6]. Here we present a case of spontaneous non-traumatic mediastinal hematoma in a patient on Imatinib therapy for GIST.

## Case presentation

**Figure 1:**
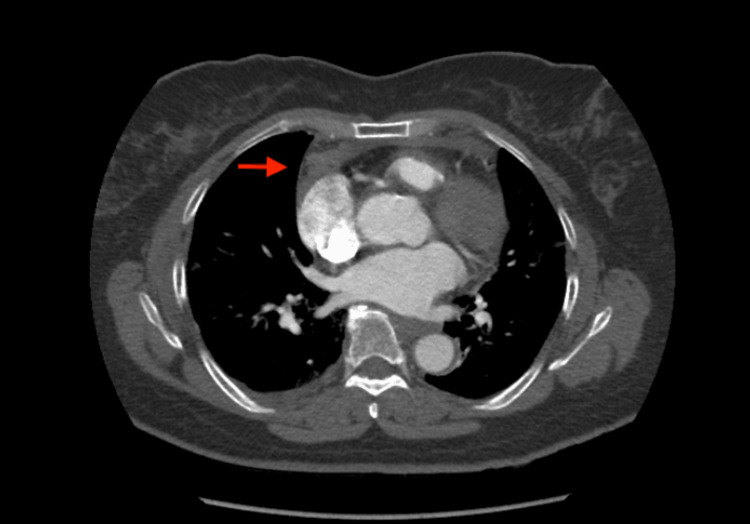
Axial view CT chest. Red arrow indicating the anterior mediastinal hematoma.

**Figure 2:**
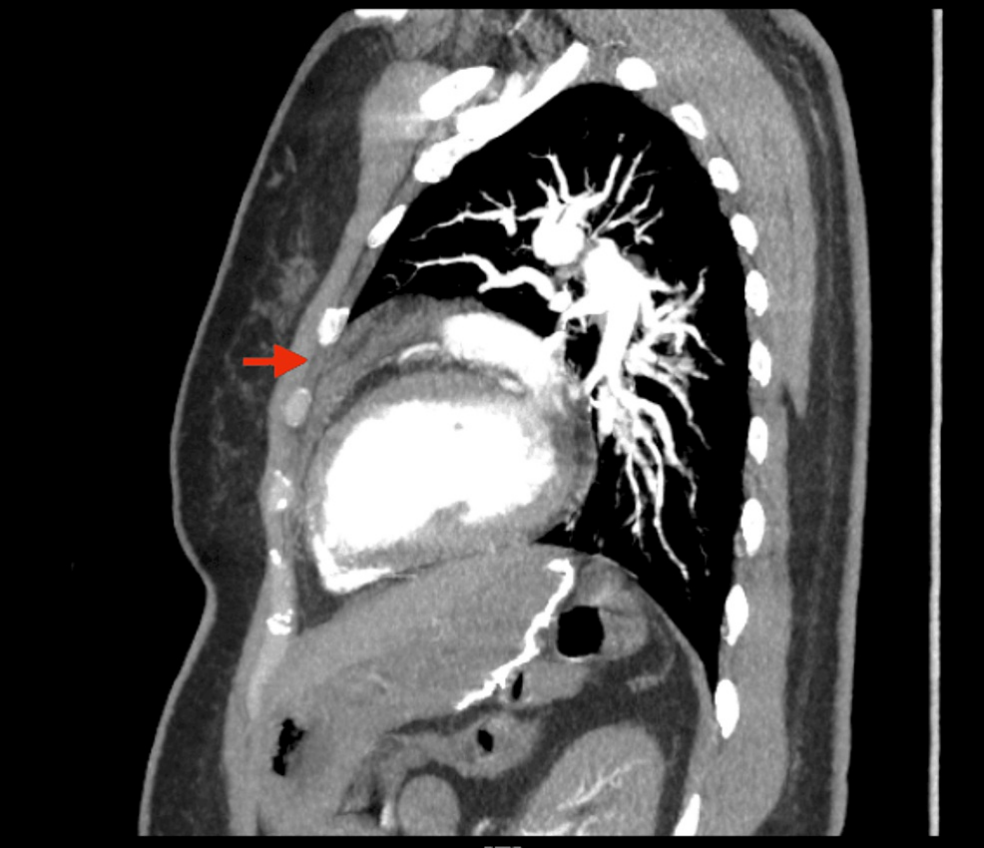
Sagittal view CT chest. Red arrow indicating the anterior hematoma within the mediastinum.

**Figure 3:**
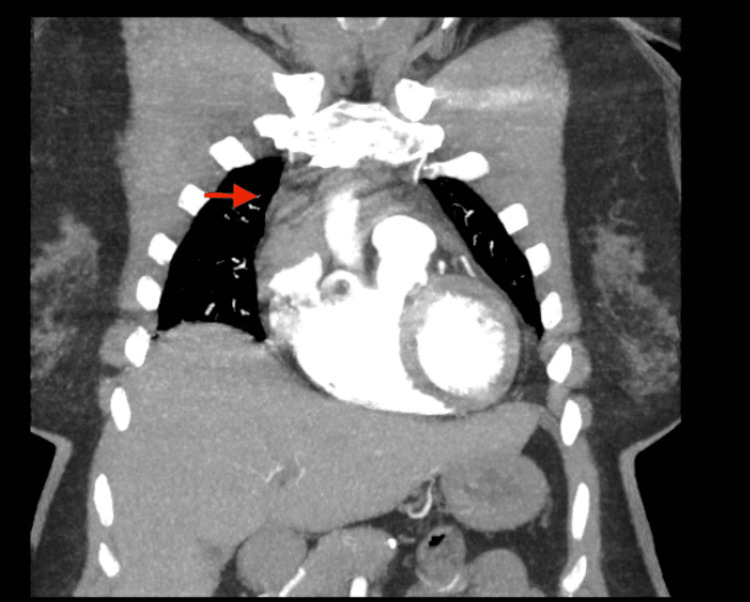
Coronal view CT chest. Red arrow indicating the anterior mediastinal hematoma.

A 67-year-old female presented to the ER with chief complaint of constant sharp right shoulder pain that progressed to her chest. The patient claimed the pain was a 10/10 and denied pain in her extremities. The patient was not on any anticoagulants. The patient also denied a history of coughing, vomiting, shortness of breath, or recent traumas. Past medical history was significant of GIST diagnosed in 2009, and no family oncologic history. The patient’s medication list was significant for Imatinib 400 mg PO daily since 2009. A cardiac workup was completed because myocardial infarction was suspected. The cardiac workup included troponin I level and EKG. However, it was non-significant. The troponin was <0.01 ng/mL, and the EKG showed normal sinus rhythm with no ST elevations. We then suspected a pulmonary embolism and requested a computed tomographic angiography scan of the chest with IV contrast. The CT scan revealed an anterior mediastinal hematoma sizing 3.9 x 2.0 x 3.5 cm (Figures 1-3).

**Figure 4:**
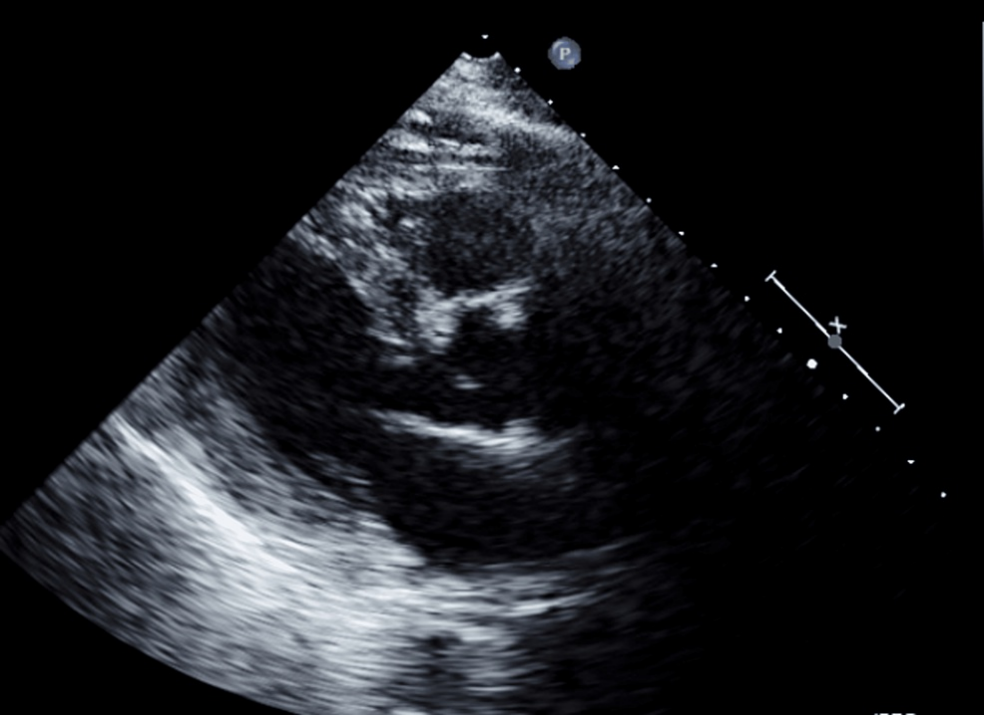
Still of echocardiography. The hematoma was not observable in the echocardiography view and was not compressing any cardiac chambers.

After the diagnosis of anterior hematoma was made, an echocardiography was ordered to determine if the hematoma was affecting cardiac function. Findings of the echocardiography illuminated normal left ventricle function and size. Mild concentric left ventricular hypertrophy was present, and the ejection fraction was within the normal range of 66% (Figure 4). As such, the echocardiography showed negligible effect of the hematoma on cardiac function. After consulting the oncologist and cardiologist, a diagnosis of non-traumatic anterior mediastinal hematoma was confirmed.

The patient was discharged and followed up by her primary care provider (PCP). The patient was educated on activity restrictions and to present to the hospital upon symptom reoccurrence. The patient was told to continue her current Imatinib therapy. Upon follow-up six months later, her chest pain had resolved, and she was exhibiting no current symptoms. Repeat CT imaging revealed complete clearance of the hematoma.

## Discussion

This patient presented with a spontaneous non-traumatic mediastinal hematoma in the absence of anticoagulant use. Most hematomas arise from some complication in the bleeding cascade or increased intrathoracic pressure, anticoagulation use, dialysis, cyst hemorrhage, trauma to the chest, or violent coughing [7]. This case was atypical since the patient did not have any of the aforementioned etiology for her mediastinal hematoma. The case allows us to expand our knowledge base on the factors that precipitate mediastinal hematomas. One factor that could have precipitated this patient's mediastinal hematoma was her concurrent use of Imatinib. Imatinib's most common side effects are GI distress (nausea, vomiting, and diarrhea) and headaches [2,8]. However, recent literature reports similar cases of bleeding with Imatinib use. In one study, two patients, one of them taking 600 mg (slightly above the normal dosage of Imatinib), developed liver hematomas and needed further blood transfusions and blood count monitoring [9]. Both patients developed liver hematomas within four months of Imatinib initiation [9]. In other studies, subdural hemorrhages have been tied to Imatinib use [4]. A total of 1.9-5.7% of intracranial hemorrhages occur with concurrent imatinib use in the absence of alternative causes [4,10,11,12]. These cases show a possible causal relationship between bleeds and Imatinib use. While most of the cases in the literature focus on liver hematomas and intracranial bleeds with Imatinib use, our case highlights a slightly different manifestation of this side effect, mediastinal hematomas. Spontaneous hematomas are rare. An anterior mediastinal hematoma linked to chemotherapy use is particularly interesting. The source of bleeding and its limited spread out of the mediastinum in our case is still a mystery. Furthermore, unlike some prior cases in the literature, our patient was never hemodynamically unstable.

## Conclusions

Our case highlights an essential point for healthcare workers to take in vigilance. Mediastinal hematomas should be within our differentials in patients using Imatinib and with chest pains to prevent delays in diagnoses and care. Furthermore, this case may demonstrate an indication for further investigation into the links between Imatinib use and the formation of mediastinal hematomas.
